# Injectable Platelet-Rich Fibrin and Hyaluronic Acid Mesotherapy for Management of Actinic Elastosis of Lower Eyelids: A Case Series

**DOI:** 10.7759/cureus.68429

**Published:** 2024-09-02

**Authors:** Sharanika A Nagaja, Rubin S John, Swetha G, Santhosh P Kumar, Murugesan Krishnan

**Affiliations:** 1 Oral and Maxillofacial Surgery, Saveetha Dental College and Hospitals, Saveetha Institute of Medical and Technical Sciences, Saveetha University, Chennai, IND

**Keywords:** facial rejuvenation, iprf, mesotherapy injection, hyaluronic acid, platelet rich fibrin

## Abstract

Rejuvenating the skin on the lower eyelids is often complicated. Treatment alternatives that have been practiced in the past had several complications. Additionally, they were not completely effective in addressing skin aging or actinic elastosis symptoms such as dark circles under the eyes. A minimally invasive therapy approach that improves the above-mentioned issues in a desirable way has been discussed in this case series. The patients selected were of the age group between 20-40 years who had actinic elastosis of the lower eyelid. The patients were injected twice at one-month intervals with a combination of injectable platelet-rich fibrin (iPRF) and hyaluronic acid. The patients were examined on the day of treatment and one month after the second injection. A progressive improvement in the esthetic outcome and a high level of patient satisfaction were observed. Apart from the predicted visible swelling right away following the iPRF injection. The outcomes have shown that a series of iPRF with hyaluronic acid injections in the lower eyelid region is a safe, proficient, pain-free, simple and rapid treatment option for actinic elastosis.

## Introduction

The eyes are often regarded as the most captivating feature of the face, playing a crucial role in perceptions of beauty, particularly among women. Their prominence underscores the importance of understanding facial anatomy, especially in the lower eyelid region, which lacks a superficial fatty layer [1]. This anatomical characteristic is significant for early detection of aging signs. Skin aging results from a complex interplay between intrinsic factors, such as genetics, and extrinsic factors, particularly UV exposure. Intrinsic aging leads to gradual degeneration of connective tissue, affecting the skin similarly to other internal organs, while extrinsic aging accelerates skin deterioration, manifesting as wrinkles and actinic elastosis, a hallmark of photoaging [2-4].

Collagen and elastin are foundational components of the skin's connective tissue, with collagen providing the structural backbone that constitutes 70% to 80% of the skin's dry weight, while elastin, though constituting only 2% to 4%, is essential for skin elasticity [5,6]. In photoaged skin, the degradation of collagen and the thickening of elastic fibers lead to solar elastosis, characterized by a dysfunctional network of elastic material [4]. To combat the visible effects of skin aging, regenerative medicine is emerging as a groundbreaking field, utilizing cell- and tissue-based therapies. One such approach involves injectable platelet concentrates, particularly platelet-rich plasma (PRP), which are increasingly recognized for their capacity to enhance tissue healing and rejuvenate the skin. However, variations in preparation methods have led to inconsistent clinical outcomes, highlighting the need for standardized protocols [5-7].

To overcome some of the limitations associated with PRP, researchers developed a second-generation platelet concentrate called platelet-rich fibrin (PRF), which is completely autologous and can be produced without anticoagulants [7,8]. PRF can exist in both solid and liquid forms, with the injectable variant, iPRF, being particularly advantageous due to its high concentration of growth factors. This formulation supports the gradual release of therapeutic agents after injection, making it an excellent option for facial rejuvenation [8]. Additionally, hyaluronic acid (HA) fillers, known for their hydrating properties and ability to provide structural support, are widely used in non-surgical aesthetic procedures, especially for the lower eyelids [9-11]. This procedure seeks to evaluate the combined effectiveness of iPRF and HA injections as a safe, minimally invasive strategy to treat both actinic elastosis and the signs of chronological aging, addressing the growing demand for effective aesthetic treatments without the need for surgery.

## Case presentation

This case series included five patients, four females and one male, ages ranging between 21 to 40 years who were treated for dark circles under the eyes, volume deficiencies, and excess skin around the lower eyelids. All patients consented to receiving the two scheduled injections. Patients with active skin lesions, acute or chronic infections, tumors or cancer, platelet functional disorders, prior eyelid surgery, prior minimally invasive procedures like botox injections or fillers in the eyelid region, and those taking anticoagulant therapy were excluded.

**Figure 1:**
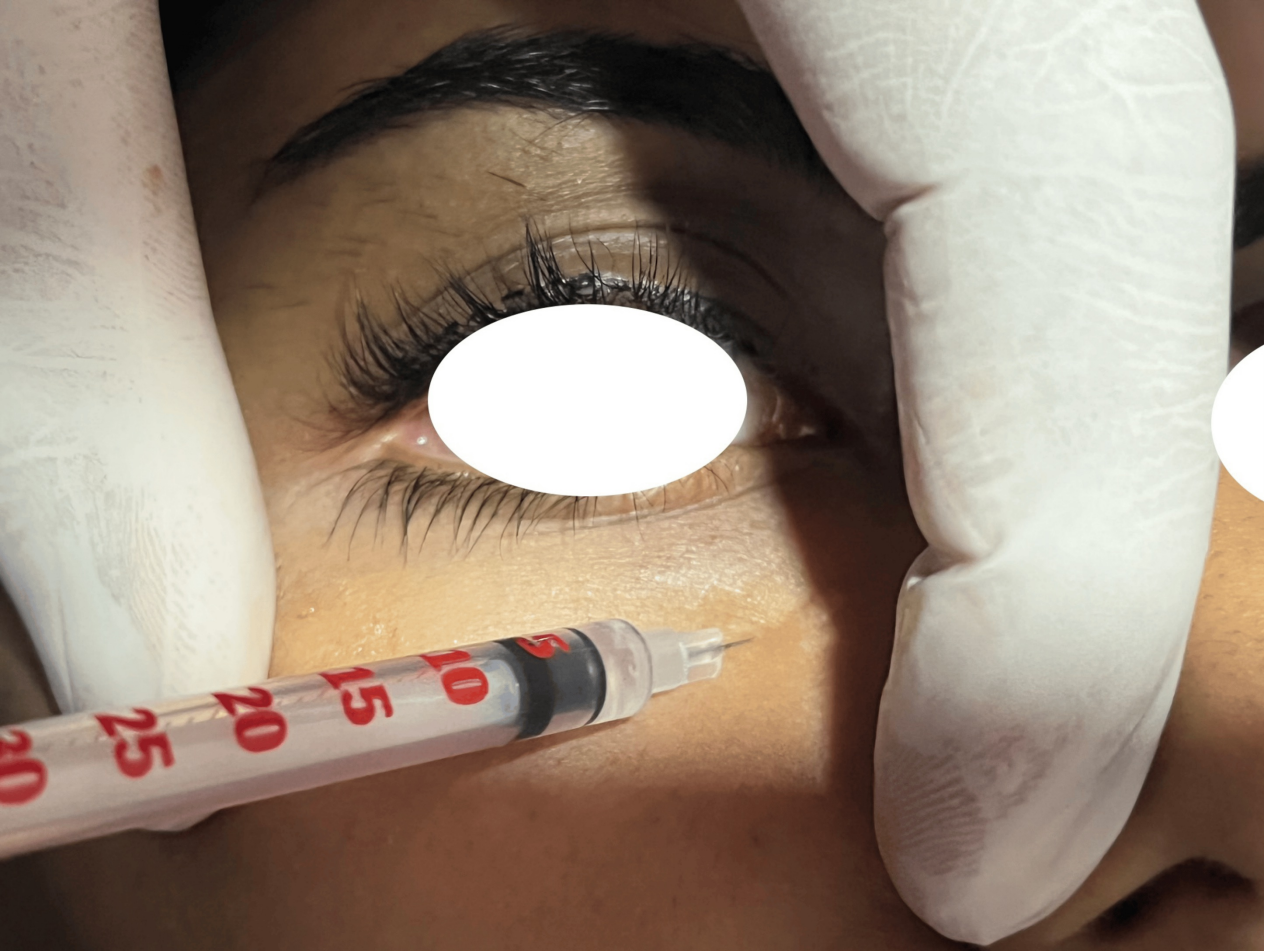
Landmarks for the injectable platelet rich fibrin (iPRF) and hyaluronic acid injection.

Each patient received two iPRF treatments at monthly intervals. At each appointment, 8 ml of the patient's peripheral venous blood was drawn via a Dispovan 10 ml syringe, placed in sterile 10 mL plastic PRF tubes without anticoagulant, and centrifuged immediately at room temperature for three minutes at 700 rpm (= 60 G RCF) using a low relative centrifugal force. An infraorbital nerve block was administered to the patient. The iPRF settled in the upper third of the syringe was drawn and mixed with HA in a 1:1 ratio into the 5 ml Dispovan syringe. A 22G cannula was inserted in the patient's lower eyelid region and 2 ml of mixture (per side) was injected intradermally, in each eye by the same investigator (Figure 1). Each participant was given the standard post-procedure instructions, which also included information on bruising, pain, swelling, and avoiding the sun. Participants were asked to rate their satisfaction before and after treatment.

**Figure 2:**
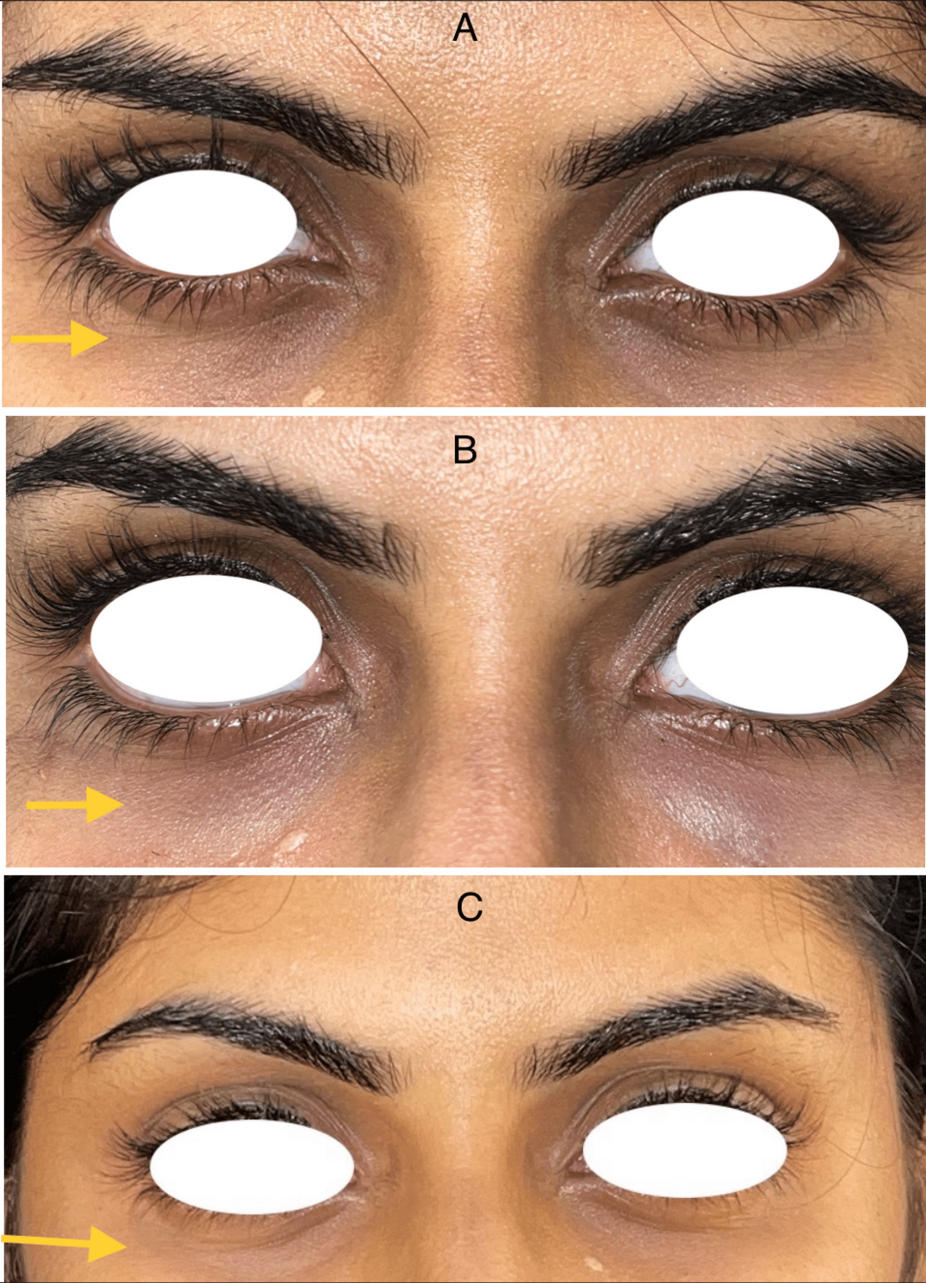
A 21-year-old female; A: Pre-operative, B: Two weeks after first infection, C: Two weeks after second injection.

**Figure 3:**
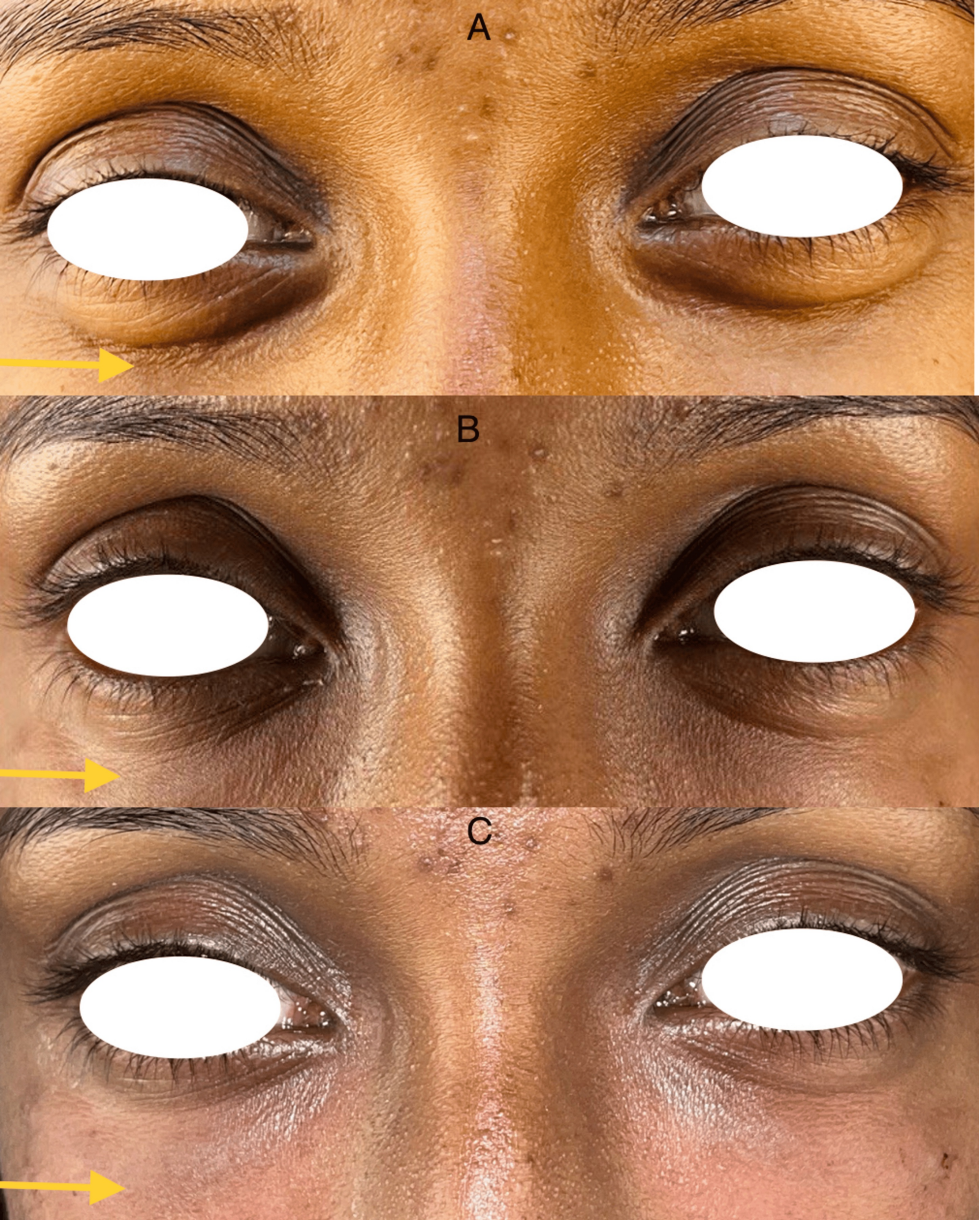
A 25-year-old female; A: Pre-operative, B: Two weeks after first infection, C: Two weeks after second injection.

**Figure 4:**
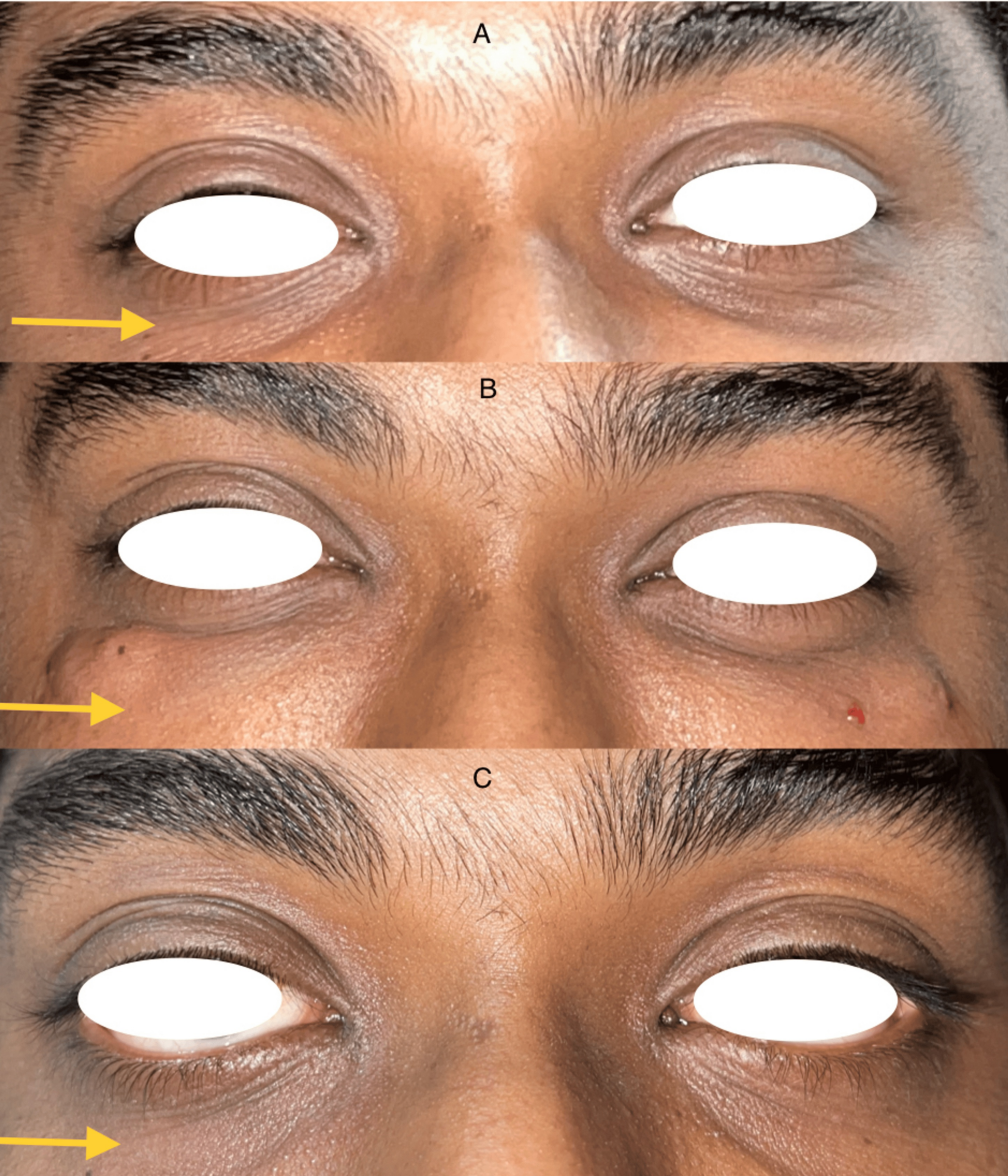
A 27-year-old male; A: Pre-operative, B: Two weeks after first infection, C: Two weeks after second injection.

Following therapy as a result of the injected volume, swelling and a slight pressure sensation were anticipated for one to two days. Although a few patients experienced distinct hematomas due to the injection's minor vein injury, no major complications developed. Regardless of age, the clinical evaluation revealed a progressive effect and overall satisfactory outcomes. With the perceived alterations in the lower eyelid region, which steadily became better from injection to injection, the patients were very satisfied with the outcome and were ready for subsequent injections (Figure 2, 3, 4). Participants were asked to rate their satisfaction before and after treatment on their appearance and treatment on a 0-3 scale: 0 = not satisfied at all, 1 = slightly satisfied, 2 = moderately satisfied, and 3 = very satisfied (Table 1).

**Table 1:** Satisfaction scale after the procedure.

SATISFACTION SCALE	Number of patients
0 (Not satisfied)	0
1 (Slightly satisfied)	0
2 (Moderately satisfied)	2
3 (Very satisfied)	3

## Discussion

The lower eyelid region presents significant challenges in treatment due to its delicate skin and complex anatomy. Common issues such as actinic elastosis and chronological aging lead to dark circles, volume deficits, and excess skin [1,2]. While minimally invasive surgical techniques exist, they often yield unsatisfactory results and carry risks, particularly because the thin skin in this area highlights color variations and low-grade inflammation. Traditional treatments like botulinum toxin can address crow's feet but do not promote tissue regeneration, resulting in limited effectiveness for aging skin. Other options, such as microneedling and fat transfers, also have drawbacks, prompting the exploration of combining injectable PRF and hyaluronic acid as an alternative approach [12-14].

Injectable PRF is a second-generation biomaterial that is completely autologous. Additionally, this product is documented to include collagen type 1 and lymphocytic growth factors. It also generates fibrin networks similar to PRF membrane. Its distinctive feature is that it remains a liquid for up to 20 minutes before fibrin polymerization occurs and a solid membrane is formed. The cellular components are dispersed throughout the three-dimensional fibrin network that makes up the structure [7,15]. This contributes to a gradual and measured release of growth factors over time, extending the duration of the effect, a characteristic similar to that of the PRF membrane. Platelets, B lymphocytes, monocytes, stem cells, neutrophils, and growth factors were discovered in injectable PRF which are also considered to be a critical part of healing along with cells and growth factors [5,8]. On the other hand, according to a study, hyaluronic acid filler is an excellent alternative to surgery without any risks of complications or recovery time for the treatment of under-eye bags or orbital fat pads. Based on these facts, we chose injectable PRF over PRP for the treatment and combined it with hyaluronic acid for actinic elastosis. Because the product contains collagen and stem cells and releases growth factors gradually over an extended period of time, it is effective for rejuvenation. Actinic elastosis, several types of alopecia, acne scars, post-laser treatments, osteoarthritis, tendinopathies, tooth extractions, infrabony defects, dental implants, jawbone lesions, and chronic wounds are potential conditions that may benefit from single or combined therapies iPRF treatment [4-7].

It is notable that the majority of the patients in our study were in their early 20s, which suggests that the lower eyelid region can be affected by aging signs even in younger age groups. In addition to the expensive surgical procedures, it is important to offer a simple and inexpensive therapy choice for this particular demographic. According to a study by Tian et al., there is a noticeable difference in the alpha granules in PRP ultrastructure between age groups [16]. Previous studies have demonstrated that platelet quantity and functionality significantly decrease with ageing [17]. According to Johnson et al., adults over the age of 40 have more extensive platelet aggregation than adults in their 20s [18]. Furthermore, Cowman et al. reported that platelet adhesion steadily increases with aging in a study of healthy volunteers in various age ranges between 20 and 89 years [19]. Since platelet function and number gradually decrease with age, platelet structure should also modify with age since platelet function and structure are significantly correlated. The majority of previous research on age-related changes in platelets was conducted on adults between the ages of 25 and 65. Numerous studies have also shown that a wide range of diseases, such as diabetes, cardiovascular disease, and various physiological conditions, have an impact on platelet ultrastructure [17,20]. 

Regarding the results, there were no discernible differences across the age groups. Evaluation of skin improvement is difficult. It is vital to standardize photographic documentation. Additionally, 3D scanners might be an appropriate instrument for precise evaluation, but their use is now limited by their expensive price.

This case series shows the benefits of the novel combination injection of iPRF and hyaluronic acid for the treatment of actinic elastosis without any adverse effects and satisfactory results, however studies on a larger scale on larger populations using different parameters can be done to standardise this procedure as treatment for actinic elastosis. This case series does not have any particular follow-up period as the development, progression and remission are subjected to multiple factors like sleep cycle, exposure to sunlight, stress, diet, etc. The improvement in all cases was observed in two weeks time. The stability of the treatment however cannot be exactly determined as the remission is largely dependent on the subjective factors and how the individual manages the same.

## Conclusions

To rejuvenate the skin and treat actinic elastosis, a combination of intradermal injection of iPRF and hyaluronic acid is a safe and effective treatment option. Patient satisfaction is excellent, and there are evident treatment results or significant side effects.
